# 

**Published:** 1908-08

**Authors:** 


### Contributors to Vol. LXIV.

### AUGUST, 1908—JULY, 1909.

Baum, Henry Clay.................................Syracuse
Brown, William Mortimer.....................Rochester
Burke, Joseph ..................................Buffalo
Colegrove. James B.........................Washington
Cottis, G. W.....................................Batavia
Conklin, W. L..................................Dansville
Gould, George M..................................Ithaca
Cumston, Charles Greene .......................Boston
Darlington, Thomas ........................New York
Davie, Neilson ..................................Glasgow
Dowd, J. Henry...................................Buffalo
Frederick. Carlton C............................Buffalo
Hayd, Herman E. .................................Buffalo
Hopkins. Henry Reed.............................Buffalo
Howe, William A.............................Phelps, N. Y.
Jones. Allen A...................................Buffalo
Jones, William B. .............................Rochester
King, James E....................................Buffalo
Kreshover, J S. ...............................New York
Levy. I. Harris ...............................Syracuse
Lewis, Bransford ..........................Saint Louis
Loughhead, W. H........................Andover. N. Y.
Loveland, B. C..................................Syracuse
MacLeod, James A...............................Buffalo
Mann. Edward C. ................................Buffalo
McGuire, Edgar R................................Buffalo
Morris. Robert T..............................New York

Morrison, A. Cressy ........................... Chicago
Munson, Edward ........................... Medina
Park, Roswell ........................... Buffalo
Parsche, Thomas W. ........................... Chicago
Pedersen, James ........................... New York
Pryor, John H. ........................... Buffalo
Putnam, James Wright ........................... Buffalo
Reed, Charles A. L. ........................... Cincinnati
Rooth, H. C. ........................... Buffalo
Senftenberg, B. F. ........................... New York
Slingerland, I. M. ........................... Fayetteville
Snively, I. Newton ........................... Philadelphia
Stocker, G. B. ........................... Buffalo
Thibaudeau, A. A. ........................... Buffalo
Toms, S. W. S. ........................... Nyack
Treves, Sir Frederick ........................... London
Ullman, Julius ........................... Buffalo
Weiss, Edward A. ........................... Pittsburg
Wende, Grover W. ........................... Buffalo
Wilcox, Dewitt G. ........................... Buffalo
Wilson, Nelson W. ........................... Buffalo
Wilson, R. J. ........................... New York

Buffalo Academy of Medicine
Medical Association of Central New York
Medical Society of the County of Erie

### BOOKS RECEIVED.

Medical Gynecology. By Samuel Wyllis Bandler, M.D., Adjunct professor of diseases of women, New York Post-Graduate Medical School and Hospital; associate attending gynecologist to the Beth Israel Hospital, New York. Octavo, pp. 676. With original illustrations. Philadelphia and London: W. B. Saunders Co. 1908. (Cloth, $5.00.)

The Natural History of Cancer, with special reference to its Causation and Prevention. By W. Roger Williams, Fellow of the Royal College of Surgeons. Octavo, pp. 519. Illustrated. New York: William Wood & Co. 1908. (Cloth, $5.00.)

Contributions to the Science of Medicine and Surgery by the Faculty in Celebration of the Twenty-fifth Anniversary, 1882-1907, of the Founding of the New York Post-Graduate Medical School and Hospital.

Transactions of the American Association of Genitourinary Surgeons. Twenty-first annual meeting held at the Shoreham Hotel, Washington, D. C., May 7, and 8, 1907, and joint session with the American Gynecological Society at the George Washington University, May 9, 1907. Vol. 2. Edward L. Keyes, Jr., secretary. New York: The Grafton Press.

Borderland Studies. By George M. Gould, M.D., Author of a Series of Medical Dictionaries, etc. Vol. 2, 12 mo, pp. 311. Illustrated. Philadelphia: P. Blakiston's Son & Co.

Golden rules of Dietetics. By A. L. Benedict, A.M., M.D., Author of Practical Dietetics. 12 mo, pp. 407. St. Louis: C. V. Mosby Co. (Cloth, $3.00.)

International Clinics. A Quarterly of Illustrated Clinical Lectures and especially prepared articles on Treatment, Medicine, Surgery, Neurology, Pediatrics, Obstetrics, Gynecology, Orthopedics, Pathology, Dermatology, Ophthalmology, Otology, Rhinology, Laryngology, Hygiene and other topics of interest to students and practitioners. Edited by W. T. Longcope, M.D., Volume II., eighteenth series. Philadelphia and London: J. B. Lippincott Co. 1908. (Cloth, $2.00.)

The Baby. Its Care and Development. For the Use of Mothers. By Le Grand Kerr, M.D., Author of Diagnostics of the Diseases of Children. 12 mo. pp. 150. Illustrated.. Brooklyn: Albert T. Huntington. 1908. (Cloth, $1.00.)

